# Direct reprogramming of urine-derived cells with inducible MyoD for modeling human muscle disease

**DOI:** 10.1186/s13395-016-0103-9

**Published:** 2016-09-15

**Authors:** Ellis Y. Kim, Patrick Page, Lisa M. Dellefave-Castillo, Elizabeth M. McNally, Eugene J. Wyatt

**Affiliations:** 1 Molecular Pathogenesis and Molecular Medicine, The University of Chicago, Chicago, USA; 2 Center for Genetic Medicine, Northwestern University Feinberg School of Medicine, 303 E. Superior St., Chicago, IL 60611 USA

**Keywords:** Urine cells, MyoD, Reprogramming, Myotubes, Muscular dystrophy, CRISPR

## Abstract

**Background:**

Cellular models of muscle disease are taking on increasing importance with the large number of genes and mutations implicated in causing myopathies and the concomitant need to test personalized therapies. Developing cell models relies on having an easily obtained source of cells, and if the cells are not derived from muscle itself, a robust reprogramming process is needed. Fibroblasts are a human cell source that works well for the generation of induced pluripotent stem cells, which can then be differentiated into cardiomyocyte lineages, and with less efficiency, skeletal muscle-like lineages. Alternatively, direct reprogramming with the transcription factor MyoD has been used to generate myotubes from cultured human fibroblasts. Although useful, fibroblasts require a skin biopsy to obtain and this can limit their access, especially from pediatric populations.

**Results:**

We now demonstrate that direct reprogramming of urine-derived cells is a highly efficient and reproducible process that can be used to establish human myogenic cells. We show that this method can be applied to urine cells derived from normal individuals as well as those with muscle diseases. Furthermore, we show that urine-derived cells can be edited using CRISPR/Cas9 technology.

**Conclusions:**

With progress in understanding the molecular etiology of human muscle diseases, having a readily available, noninvasive source of cells from which to generate muscle-like cells is highly useful.

**Electronic supplementary material:**

The online version of this article (doi:10.1186/s13395-016-0103-9) contains supplementary material, which is available to authorized users.

## Background

The genetic diversity of muscle diseases, including the muscular dystrophies, is extensive [1, 2]. Defining underlying mechanisms and developing therapies to correct these defects require appropriate models of disease. Animal models offer in vivo insight and have proved highly useful, but animal models are time consuming and costly to develop and cannot represent the large number of alleles responsible for muscle diseases. Cellular models of disease have expanded in recent years with significant advances in reprogramming and the creation of induced pluripotent stem cells (iPSCs) for many disorders. For muscle diseases, the comparative ease of generating cardiomyocytes, rather than skeletal myocytes, from iPSCs has led to this cell type being used as a surrogate for skeletal muscle [3–5]. Although progress has been made in generating muscle-like lineages from iPSCs, this process is still time-consuming and has variable outcomes within and across culture systems [6, 7]. Other commonly used cell models of skeletal muscles include the mouse C2C12, rat L6, and human myoblast cell lines, which undergo differentiation into multinucleate myotubes under appropriate conditions [8, 9]. These myoblast models have been highly useful for the field but represent normal skeletal muscle and not disease-specific conditions. While myoblasts can be cultured from human muscle, biopsies from patients are invasive and at times problematic for those with muscle mass-limiting conditions. A noninvasive patient-specific in vitro skeletal muscle model is desirable for studying pathogenesis and testing potential drugs.

Direct reprogramming of fibroblasts with MyoD into myotubes was a central experiment used to demonstrate the role of transcription factors [10, 11]. Since that original observation, MyoD delivery into fibroblasts has been optimized using viral vectors and this method can be applied to make directly reprogrammed myogenic cells from humans and mice [12, 13]. A fusion between MyoD and the estrogen receptor was generated to create a tamoxifen-inducible MyoD, and this construct was used in mouse and human fibroblasts to induce myogenesis [14–16]. Thus, direct reprogramming of fibroblasts remains a highly useful method for modeling human muscle diseases. However, dermal fibroblasts are obtained through skin biopsy, and the invasive nature of skin biopsy, while considerably less than muscle biopsy, can be particularly problematic in children.

One potential noninvasive source of patient-specific cells is cells isolated from urine. It has been estimated that about 2000 to 7000 renal tubular epithelial cells are shed in urine everyday [17, 18]. Obtaining a urine sample is noninvasive, making urine a good source of cells, especially from children. Urine cells have been isolated from both healthy volunteers and patients of a wide age range [18–21]. Urine-derived cells (UDCs) are thought to be of a mixed population that originates from either the renal epithelium or the uroepithelium [18, 21]. Interestingly, a subset of UDCs has been observed to carry mesenchymal stem cell markers as well as low endogenous expression of Oct3/4, a pluripotency marker [4, 20, 22]. As such, UDCs demonstrate propensity for differentiation into several lineages, one of which is muscle-like [20, 23].

We show here that UDCs serve as a noninvasive source for MyoD-induced myogenesis. UDCs transduced with a tamoxifen-inducible MyoD lentiviral vector (iMyoD) formed multinucleate myotubes following induction with tamoxifen and culture in differentiation media for up to 35 days. Urine cell-derived myotubes expressed muscle marker transcripts, formed sarcomeres, and displayed contractile properties. Further, UDCs isolated from individuals with muscular dystrophies were also able to form myotube-like structures and reflected the disease mutations. Thus, UDCs are a ready source for direct reprogramming by MyoD and provide an easily obtainable, noninvasive source for modeling disease mechanisms.

## Methods

### Urine collection and cell isolation

Written and informed consent was obtained from all human subjects. All work was approved by and conducted under the Institutional Review Boards of the University of Chicago (IRB8249) and Northwestern University (IRB STU00104548), which serve as the ethics boards for each institution, respectively. All studies were conducted in compliance with the Helsinki Declaration. Urine samples were collected using midstream clean catch kits according to the directions provided in the kits (Parter Medical Products Inc, Carson, CA; 273516). Cells were isolated within 1 h of collection using a protocol previously published with minor modifications [24]. Briefly, the entire urine samples were equally distributed into 50-mL conical tubes and centrifuged at 400×*g* for 10 min at room temperature. The supernatant was aspirated leaving ~1 mL of urine into which pellets were resuspended and combined into a single tube, if necessary. Ten milliliters of wash buffer was added per 100 mL of initial urine sample. Samples were centrifuged at 200×*g* for 10 min at room temperature. The supernatant was aspirated leaving ~0.2 mL, and the cell pellet was resuspended in 1 mL of primary media. All media formulations were obtained from a previously published protocol and are detailed below [24]. Cells were plated in 24-well plates pre-coated with 0.1 % gelatin (Millipore, Billerica, MA; ES-006-B, Stemcell Technologies, Vancouver, Canada; 7903). Roughly one third of the cell suspension was plated in the first well, with the remaining two thirds equally divided into four additional wells. The final volume in each well was then brought to 500 μL with primary media. The plates were placed in a 37 °C incubator with 5 % CO_2_.

For 3 days, 500 μL of primary media was added to each well every 24 h. On day 4, 1.5 mL of primary media was removed and replaced with 500 μL of proliferation media. An aliquot of the primary media was added to a separate dish containing Dulbecco’s Modified Eagle Medium (DMEM) supplemented with 10 % FBS without antibiotics or antimycotics to test for potential contamination. On day 5, all media were removed from each well and replaced with 500 μL of proliferation media, which was changed daily until the isolated cells expanded and were replated in larger dishes. Antibiotics and antimycotics were removed from media once uncontaminated cultures were confirmed. Isolated cells were observed as early as 1 day after the addition of proliferation media. When the cells became confluent or when cell foci began to outgrow the monolayer, cells were trypsinized using 0.25 % trypsin-EDTA (Thermo Fisher Scientific, Waltham, MA; 25200-072), subcultured, and designated as passage 1 (p1). Modifications from [24] include plating of cells in five wells of a 24-well gelatin-coated plate (vs a single well of 12-well plate), increase of FBS content in the proliferation media to 15 %, and the removal of the antimycotics and antibiotics from the media after lack of contamination was observed.

### Media composition

All media were made following a previously published protocol with the following modifications [24]. Wash buffer consisted of 1× phosphate-buffered saline (PBS) without Ca^2+^ and Mg^2+^ (Thermo Fisher Scientific, Waltham, MA; 14190-250) supplemented with 1 % penicillin/streptomycin (Thermo Fisher Scientific, Waltham, MA; 15070-063) and 0.5 μg/mL amphotericin B (Sigma Aldrich, St. Louis, MO; A2942). Primary media were composed of 1:1 mix of high glucose DMEM without sodium pyruvate (GE Healthcare, Logan, UT; SH30022.FS) and Ham’s F-12 Nutrient Mix (Thermo Fisher Scientific, Waltham MA; 11765-054) supplemented with Renal Epithelium Growth Medium SingleQuot Kit Supplements (Lonza, Basel, Switzerland; CC-4127), 10 % fetal bovine serum (Thermo Fisher Scientific, Waltham, MA; 16000-044), 1 % penicillin/streptomycin, and 0.5 μg/mL amphotericin B. Proliferation media were composed of 1:1 mix of Renal Epithelium Growth Medium Bullet Kit (Lonza, Basel, Switzerland; CC-3190) and high glucose DMEM supplemented with 15 % FBS, 0.5 % Glutamax (Thermo Fisher Scientific, Waltham, MA; 35050-061), 0.5 % nonessential amino acids (Thermo Fisher Scientific, Waltham, MA; 11140-050), and 2.5 ng/mL of bFGF (Peprotech, Rocky Hill, NJ; 100-18B, Miltenyi Biotec Inc, San Diego, CA; 130-093-842), PDGF-AB (Peprotech, Rocky Hill, NJ; 100-00AB), and EGF (Peprotech, Rocky Hill, NJ; AF-100-15). The Renal Epithelium Growth Medium (REGM) Bullet Kit was made according to the manufacturer’s instructions, with the omission of the amphotericin B/gentamycin supplement. Freeze media were composed of DMEM (Thermo Fisher Scientific, Waltham, MA; 11995-073) supplemented with 30 % FBS, 1x pen/strep, and 10 % DMSO (Sigma Aldrich, St. Louis, MO; D2650).

### Lentiviral construct and transduction

The tamoxifen-inducible MyoD lentiviral construct (pLv-CMV-MyoD-ER(T)), referred to as iMyoD, was previously described [14] and kindly provided by Dr. Jeffrey Chamberlain (University of Washington; Addgene plasmid # 26809). The construct was packaged by the Skin Disease Research Center DNA/RNA Delivery Core at Northwestern University (Chicago, IL). Urine cells from healthy volunteers or patients were plated in 12-well plates at 75,000 cells/well and transduced within 24–36 h of plating with iMyoD at MOI = 50. Transductions were performed in proliferation media with reduced serum (5 %) and 7.5 μg/mL polybrene (Millipore, Billerica, MA; TR-1003-G) for 6–16 h in a 37 °C incubator.

### Evaluation of transduction efficiency

After transduction, an aliquot of cells were plated in two wells of a 24-well plate at 2000–10000 cells/cm^2^. Cells were treated with 5 μM 4-hydroxytamoxifen (Sigma Aldrich, St. Louis, MO; H7904) for 24 to 48 h, and MyoD was detected using immunofluorescence. Methods and antibodies are as described below. A minimum of three fields and a minimum of 100 cells total were analyzed to determine transduction efficiency. MyoD-positive nuclei and total nuclei were counted per field, and efficiency was calculated by dividing the number of MyoD-positive nuclei by the total number of nuclei per field. Efficiencies were graphed as mean ± standard error of the mean (SEM) using GraphPad Prism (GraphPad, La Jolla, CA). All images were taken using the Floid Cell Imaging Station (Thermo Fisher Scientific, Waltham, MA; 4471136). The cell counts for MyoD-positive nuclei were performed manually using ImageJ (NIH, Bethesda, MD), while the cell counts for total nuclei were performed using the Particle Analyzer function of ImageJ (NIH, Bethesda, MD).

### Myogenic differentiation

Transduced cells were plated in 24-well plates coated with 8 μg/cm^2^ collagen I (Sigma Aldrich, St. Louis, MO; C3867, Corning, Corning, NY; 354236) at a density of 37,500 to 50,000 cells/cm^2^ and cultured until confluent. Cells were then overlaid with Growth Factor Reduced Matrigel (Corning, Corning, NY; 354230) diluted 1:4 in cold DMEM/F-12 50:50 mix (Corning, Corning, NY; 10-092-CV). Matrigel was allowed to gel for 2 to 3 hours at 37 °C. The Matrigel-overlaid iMyoD-treated cells were induced with 4-hydroxytamoxifen (2.5 μM, 24–48 h) diluted in high glucose DMEM (Thermo Fisher Scientific, Waltham, MA; 11995-073) supplemented with 10 % FBS. Hydrocortisone dexamethasone (HD) differentiation media were based on previously published formulation [23, 25] and were composed of high glucose DMEM with sodium pyruvate supplemented with 10 % FBS, 5 % horse serum (Thermo Fisher Scientific, Waltham, MA; 16050-122), 50 μM hydrocortisone (Sigma Aldrich, St. Louis, MO; H0888), and 0.1 μM dexamethasone (Sigma Aldrich, St. Louis, MO; D4902). Two hundred and fifty microliters of differentiation media was replaced every 72 h. Additional coatings tested during plating optimization included collagen IV (Sigma Aldrich, St. Louis, MO; C5533), fibronectin (Sigma Aldrich, St. Louis, MO; F1141), laminin (Sigma Aldrich, St. Louis, MO; L2020), Growth Factor Reduced Matrigel, poly-l-lysine (Sigma Aldrich, St. Louis, MO; P4832), and poly-d-lysine (Sigma Aldrich, St. Louis, MO; P6407).

### RNA analysis

Differentiated myotubes were collected at three different time points (7, 14, and 28 days). Cells were gently washed with cold 1× PBS. Washes were repeated until most of the Matrigel was removed. RNA was isolated in TRIzol (Thermo Fisher Scientific, Waltham, MA; 15596-018) following the manufacturer’s instructions. Glycogen (Thermo Fisher Scientific, Waltham, MA; AM9510) was added at 50–100 μg/mL to the isopropanol before RNA precipitation. RNA concentration was determined using NanoDrop 2000 (Thermo Fisher Scientific, Waltham, MA). RNA (1000–1500 ng) was reverse transcribed using qScript cDNA SuperMix (Quanta Biosciences, Gaithersburg, MD; 95048-025). Complementary DNA (cDNA) (30–50 ng) was used per PCR reaction, and the products were separated on 1.5 % agarose gel with ethidium bromide. Gels were visualized and imaged on the UVP Transilluminator (UVP, Upland, CA). Quantitative PCR was performed with 30–50 ng of cDNA using iTaq Universal SYBR Green Supermix (Bio-Rad, Hercules, CA; 1725124) with biological triplicates when available and technical duplicates. Cq values were averaged for each pair of technical duplicates and normalized to GAPDH. ΔCq values were calculated as Cq_(gene of interest)_—Cq_(GAPDH)_, and ΔΔCq values were calculated as 2^(−ΔCq). ΔΔCq values were then multiplied by 1000 as an arbitrary factor for graphing purposes. ΔΔCq values were graphed as mean ± SEM using GraphPad Prism (GraphPad, La Jolla, CA). Primers used for RT- and qRT-PCR are listed in Tables 1 and 2.

**Table 1:** Primers for PCR

*ACTN2*	F: CTAAAATGTTGGATGCTGAAGACA
	R: CAGCAATATCCGACACCATCTTGC
*DES*	F: AAGGGCACTAACGATTCCCT
	R: CATCCCGTGTCTCGATGGTC
*DMD* (exons 1 to 3)	F: TCCTGGCATCAGTTACTGTGTT
	R: TATGCTGCTTCCCAAACTTAGAA
*DMD* (exons 42 to 50/51)	F: CAATGCTCCTGACCTCTGTG
	R: GAGTAGGAGAGGCTCCAATA
*DMD* (exons 44 to 48)	F: GAACAGTTTCTCAGAAAGAC
	R: GCAGCAGATGATTTAACTGC
*GAPDH*	F: ACCACAGTCCATGCCATCAC
	R: CCACCACCCTGTTGCTGTAG
*MYH2*	F: GAGGCTGACTCGTCCTGCTTTA
	R: GACTGATTCTCTCGGTCAGTCA
*MYH3*	F: CTTGTGGGCGGAGGTCTGG
	R: GCCACTTGTAGGGGTTGACA
*MYOG*	F: ACCCAGGGGATCATCTGCTCA
	R: CACTGGCATCGGGAAGAGAC
*SGCG*	F: TCTAAGATGGTGCGTGAGCAG
	R: GCCACAGACAGGTACAGCTT

**Table 2:** Primers for qPCR

*ACTN2*	F: CTAAAATGTTGGATGCTGAAGACA
	R: CATTCCAAAAGCTCACTCGCTA
*DES*	F: GATCAATCTCCCCATCCAGA
	R: TGGCAGAGGGTCTCTGTCTT
*GAPDH*	F: GTGGACCTGACCTGCCGTCT
	R: GGAGGAGTGGGTGTCGCTGT
*MYH2*	F: TCTCCAAAGCCAAGGGAAAC
	R: TGCGCAGTCAGGTCATTGAT
*MYH3*	F: TTGATGCCAAGACGTATTGCT
	R: GGGGGTTCATGGCGTACAC
*MYOG*	F: GCTGTATGAGACATCCCCCTA
	R: CGACTTCCTCTTACACACCTTAC

### Immunofluorescence microscopy

Differentiated myotubes were subject to immunofluorescence microscopy at four different time points (7, 14, 28, and 35 days). Myotubes were gently washed with 1× PBS (Thermo Fisher Scientific, Waltham, MA; 14200-166) three times, or until most of the Matrigel was removed, and then fixed in either 4 % paraformaldehyde (15 min at RT) or ice cold 100 % methanol (2 min on ice). Myotubes fixed in paraformaldehyde were subsequently permeabilized in 0.25 % Triton-X (Sigma Aldrich, St. Louis, MO; T8787) for 20 min at RT. Cells were blocked in 10 % horse serum for 30 min to 1 h at 4 °C. Primary antibody incubations were done overnight at 4 °C. Cells were washed three times for 10 min each with PBS, where PBS was supplemented with 0.1 % Triton-X in the second wash. Cells were incubated with secondary antibodies (1 h at RT) and then washed three times for 10 min each as described above. Nuclei were stained with Hoechst 33342 (1:10,000, Thermo Fisher Scientific, Waltham, MA; H3570). Plates were imaged on the Zeiss Axio Observer Z.1 inverted microscope (Carl Zeiss Microscopy, Jena, Germany) and/or Floid Cell Imaging Station. All antibodies were diluted in 0.1 % Triton-X and 2 % horse serum, and all solutions were made with 1× PBS.

### Antibodies

Primary antibodies were as follows: rabbit polyclonal anti-MyoD (1:1000, Santa Cruz, Dallas, TX; sc-304), mouse monoclonal anti-desmin (1: 1000, Sigma Aldrich, St. Louis, MO; D1033), mouse monoclonal anti-α-actinin (1: 1000, Sigma Aldrich, St. Louis, MO; A7811), rabbit polyclonal anti-dystrophin (1: 1000, Thermo Fisher Scientific, Waltham, MA; PA1-37587), mouse monoclonal anti-dystrophin (1: 100, Leica Biosystems, Newcastle, UK; NCL-DYSB), mouse monoclonal anti-MYH1 (1: 10 or 2 μg/mL, deposited to Developmental Studies Hybridoma Bank by Fischman, DA; MF 20), mouse monoclonal anti-fast myosin heavy chain (1: 1000, Sigma Aldrich, St. Louis, MO; M4276), mouse monoclonal anti-titin antibody (1: 10 or 2 μg/mL, deposited to Developmental Studies Hybridoma Bank by Greaser, ML; 9 D10), and rabbit polyclonal anti-γ-sarcoglycan (1: 300 to 1: 500 [26]). Secondary antibodies were as follows: Alexa Fluor 488 donkey anti-rabbit (1: 1,1000; A21206), Alexa Fluor 488 donkey anti-mouse (1: 1000; A21202), Alexa Fluor 594 donkey anti-rabbit (1: 1000; A21207), and Alexa Fluor 594 donkey anti-mouse (1: 1,1000; A21203, all Thermo Fisher Scientific, Waltham, MA).

### Sequencing

*DMD* exons 42 through the junction of exons 50 and 51 were amplified by PCR, gel purified using QIAEXII Gel Extraction Kit (Qiagen, Valencia, CA; 20021). The purified products were amplified by PCR using nested primers for *DMD* exons 44 through 48 and then sequenced at the NUSeq Core at Northwestern University (Chicago, IL). Chromatograms and sequences were analyzed on SeqMan Pro (DNASTAR, Madison, WI) and FinchTV (Geospiza, Seattle, WA).

### CRISPR/Cas9 design

To generate a point mutation in control urine cells, we used the Cas9-GFP plasmid designed by the Zhang lab (Addgene plasmid #48138) [27]. The CRISPR design tool (crispr.mit.edu) was used to identify and select guide RNA (gRNA) targets in human *SGCG* exon 6. The target on the anti-sense strand was gRNA 5′ CTAAGGTCTTGAAACGGGT 3′. This guide permitted a synonymous mutation of the protospacer adjacent motif (PAM) and preserved the 5′ *SGCG* exon 6 sequence. The gRNA was expressed from the U6 polymerase III promoter as previously described [28]. The promoter, crRNA-tracrRNA, and target were synthesized as a gBlock by Integrated DNA Technologies (IDT, Coralville, IA), then PCR amplified (F: TGTACAAAAAAGCAGGCTTTAAAG; R: TAATGCCAACTTTGTACAAGAAAG), and purified using the Qiagen PCR purification kit (Qiagen, Valencia, CA; 28106). The single-stranded oligodeoxynucleotide (ssODN) was designed according to published guidelines [29]. The ssODN, based on the anti-sense strand template, was centered in between the nuclease site and the desired thymine deletion with 71 bp flanking arms on either side (142 bp total). It contained a downstream G > A synonymous mutation that destroyed the PAM –NGG site (CGG > CAG). The ssODN ultramer ggagaggttgttaatatttgaacaaaaattcttacCTAAGGTCTTGAAACGGGTCaGCTCTGACAAGGGGTGTCTCCACTGAATGTTC*AAAA*GAGCCCCTTCAGGCCctaaacaaaaaacaaagatgtatcaggagcagaag was synthesized and PAGE purified by IDT. Uppercase letters indicate the *SGCG* exon 6 coding region, and lowercase letters indicate the flanking intronic regions. The single underlined region encodes the targeted mutation of the protospacer adjacent motif (PAM), and the italicized sequence is the 4 adenine (AAAA) sequence which encodes the single thymine (T) deletion in the exon 6 coding region.

### Electroporation with CRISPR reagents

On the day of electroporation, urine cells were trypsinized, centrifuged at 400×*g* for 5 min (RT), and resuspended in PBS. An aliquot was removed for cell counting, and cells were centrifuged at 400×*g* for 5 min (RT). Cells were resuspended in electroporation buffer 1 × 10^6^ cells/mL (Bio-Rad, Hercules, CA; 165-2677) and 2 × 10^5^ cells were added to the Cas9 GFP plasmid (1 μg), U6 gRNA (500 ng), and ssODN (0.5 nmol) reagents. After gentle mixing, the cells were placed in a sterile cuvette with a 0.4-cm gap (Bio-Rad, Hercules, CA; 1652088) and electroporated using Gene Pulser Xcell Electroporation System (Bio-Rad, Hercules, CA; 1652660) with the following settings: (1) single square wave pulse, 200 V, 20 msec or (2) three square waves pulses, 150 V, 20 msec (0.1 s between pulses). The electroporated cells were added to gelatin-coated six-well plates and brought to a final volume of 2 mL with proliferation media. The media were replaced within 12 h and replaced every 24 h for 3–5 days until fluorescence-activated cells sorting (FACS).

### FACS sorting and clonal expansion

Prior to cell sorting, 96-well plates were pre-coated with 0.1 % gelatin and placed in the incubator for 1 h. Gelatin was removed and replaced with 100 μL of proliferation media that were supplemented with 0.5× pen/strep. The electroporated cells were trypsinized, centrifuged, and resuspended in 1 mL of proliferation media supplemented with 0.5× pen/strep. Single cell sorting of GFP-positive cells into 96-well plates was conducted by the Northwestern Flow Cytometry Facility using the FACS AriaII (BD Biosciences, San Jose, CA). After sorting, 96-well plates were briefly centrifuged at 200×*g* for 1 min (RT) and placed in a 37 °C incubator. After 48 h, 100 μL of proliferation media without pen/strep was added to the 96-well plates. A complete media exchange was performed on days 4, 8, and 12 using proliferation media without pen/strep. Expansion of single cell clones was evident 7–10 days after FACS, with confluent wells observed about day 14. Confluent wells were subcultured onto gelatin-coated 24-well plates until confluent and then subcultured onto gelatin-coated six-well plates.

### Genotype analysis of clones

Clones expanded to the six-well stage were analyzed for genome editing via the CRISPR/Cas9 system. Genomic DNA was isolated and PCR amplified using the GeneArt cleavage detection kit (Thermo Fisher Scientific, Waltham, MA; A24372) according to the manufacturers’ instructions. The primer set amplified a genomic region containing *SGCG* exon 6 along with a portion of the flanking introns (F: TAGGGAAAGAGTTGGGCCTATTT) (R: GAGGCAGATTGGAGAGGAAGAA). To test for clones edited through homology-dependent repair (HDR), PCR-amplified products were subjected to Alu1 digest and restriction fragment length polymorphism (RFLP) analysis. In addition, PCR products from individual clones were sequenced at the NUSeq Core at Northwestern University (Chicago, IL). Chromatograms and sequences were analyzed using SeqMan Pro (DNASTAR, Madison, WI).

## Results

### Generation of urine-derived cells with inducible MyoD expression

**Fig. 1:**
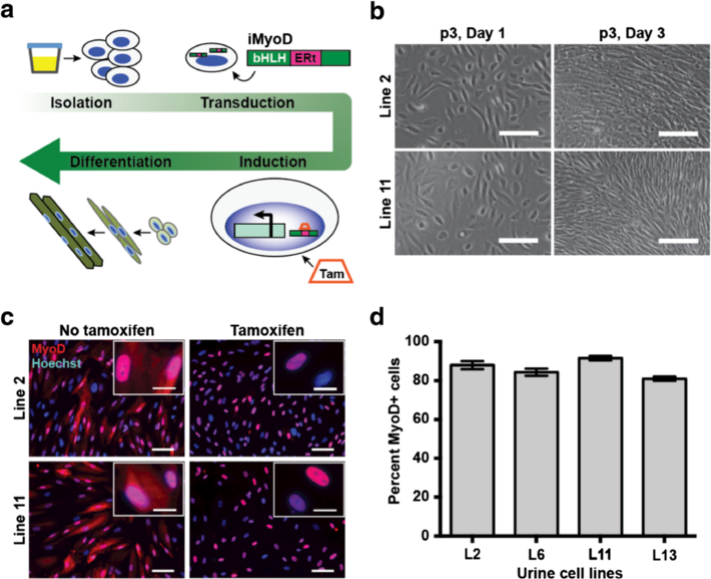
Transduction of urine-derived cells (UDCs) with an iMyoD lentiviral construct for myogenic reprogramming. **a** General overview for generating in vitro model of human skeletal muscle using UDCs and iMyoD construct. The cells were first isolated from urine samples and then transduced with lentivirus encoding an inducible MyoD. The construct includes a 4-hydroxytamoxifen (tamoxifen, TAM) response element from the estrogen receptor (ERt) downstream of the basic helix loop helix (bHLH) DNA binding domain of MyoD. After induction with tamoxifen, cells were placed in differentiation media. **b** Bright-field images of isolated urine cell cultures at passage 3. *Scale bar* = 25 μm. **c** Images of immunolabeled MyoD (*red*) in UDCs after transduction with the iMyoD lentivirus, and iMyoD-transduced urine cells treated with tamoxifen (5 μM, 48 h). Nuclei were marked with Hoechst 33342 (*blue*). *Scale bar* = 100 μm. Inset images show increased MyoD localization to the nuclei after tamoxifen. *Inset* of tamoxifen-treated line 2 cells shows one nuclei with MyoD localization (*left*) and one without (*right*). *Inset scale bar* = 25 μm. **d** Lentiviral iMyoD transduction efficiency across four control urine cell lines, shown as the percent of MyoD-positive cells relative the total number of cells

Urine-derived cells were previously shown to be a useful source for the generation of iPSCs [30]. To test direct reprogramming into myogenic lineages using urine cells, we used viral delivery of the muscle transcription factor MyoD. We used a previously characterized tamoxifen-inducible MyoD (iMyoD) lentivirus, which permits the culture and expansion of cells in an undifferentiated state until a reprogramming start point is determined [14]. The procedure includes urine cell isolation, proliferation, iMyoD transduction, tamoxifen induction, myogenic differentiation, and evaluation (Fig. 1a). Urine was isolated from multiple healthy individuals (*n* > 8), and urine cells were isolated and cultured as described [22, 24]. Bright-field images from two typical urine cultures demonstrated characteristic urine cell morphology and growth (Fig. 1b). More than one cellular morphology was noted, and independent cultures from the same individual were observed to have differential growth rates. In general, we selected clones demonstrating rapid expansion and displaying a type 1 morphology, characterized as a monolayer of polar cells with a “rice-grain” appearance, that maintained close cell-cell contact [19, 31, 32]. Both fresh and freeze-thawed urine cells were used for lentiviral transduction with success.

Urine cells were transduced with the iMyoD lentivirus, regularly achieving >80 % efficiency with an MOI of 50, as determined by immunofluorescence microscopy and image analysis for MyoD expression (Fig. 1c, d). In the absence of tamoxifen, MyoD was seen in cytoplasm. After tamoxifen exposure, MyoD staining also became nuclear (Fig. 1c). Because lentiviral transduction was of such high efficiency, nuclear MyoD staining was routinely evident in 80 % of the cultures (Fig. 1d).

### Morphological and molecular myogenic reprogramming of iMyoD urine-derived cells

**Fig. 2:**
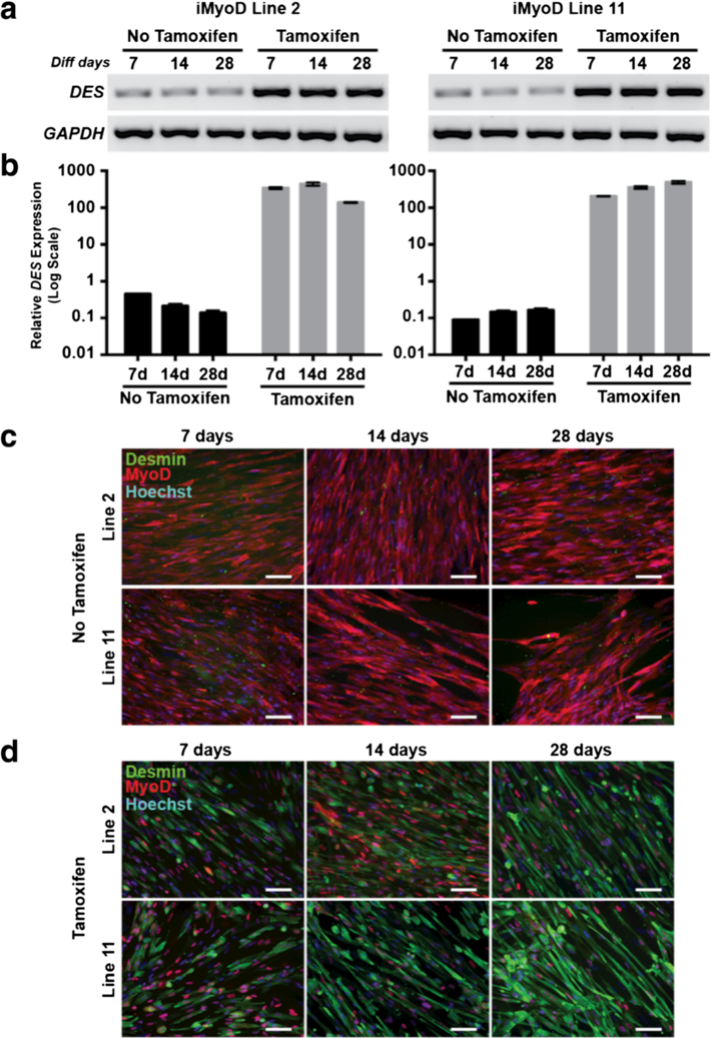
Tamoxifen-induced myogenic reprogramming of iMyoD-transduced UDCs. **a** RT-PCR time course evaluation of *DES* (desmin) mRNA expression in two UDC lines (2 and 11). iMyoD-transduced cells were treated with or without tamoxifen (2.5 μM, 24 h) and then cultured in differentiation media as indicated (7, 14, and 28 days). *GAPDH* mRNA expression is shown as a control. **b** Real-time semi-quantitative PCR results for *DES* mRNA expression in urine cell lines 2 and 11. **c** Immunofluorescence microscopy of MyoD (*red*) and desmin (*green*) protein expression in iMyoD-transduced control lines 2 and 11, without tamoxifen treatment. In the uninduced state, MyoD demonstrated a cytoplasmic distribution, and no appreciable desmin expression was observed. Nuclei (*blue*); *Scale bar* = 100 μm. **d** After induction, MyoD (*red*) became more nuclear concomitant with cytoplasmic desmin (*green*) protein expression. Myotube elongation progressed over time in culture. *Scale bar* = 100 μm

After iMyoD transduction, urine cells underwent testing for myogenic reprogramming with or without tamoxifen followed by culture in differentiation media (HD media), a formulation previously shown to enhance the endogenous myogenic potential of untransduced urine cells [23]. RT-PCR amplification showed upregulation of the *DES* (desmin) transcript in tamoxifen-treated cells by the 7-day (7d) time point, and this was sustained throughout the differentiation time course (Fig. 2a). Low-level *DES* expression was detected in uninduced iMyoD urine cells cultured in HD media alone (Fig. 2a, b). Immunofluorescence microscopy showed that desmin protein expression was evident in tamoxifen-induced iMyoD cells at the earliest time point (7d) and increased with extended culture in HD differentiation media (Fig. 2c, d). Desmin protein expression was observed concomitant with increased myotube organization and elongation (Fig. 2c, d). Desmin protein expression was not readily evident in uninduced iMyoD cells (Fig. 2c).

### Myogenic differentiation and maturation

**Fig. 3:**
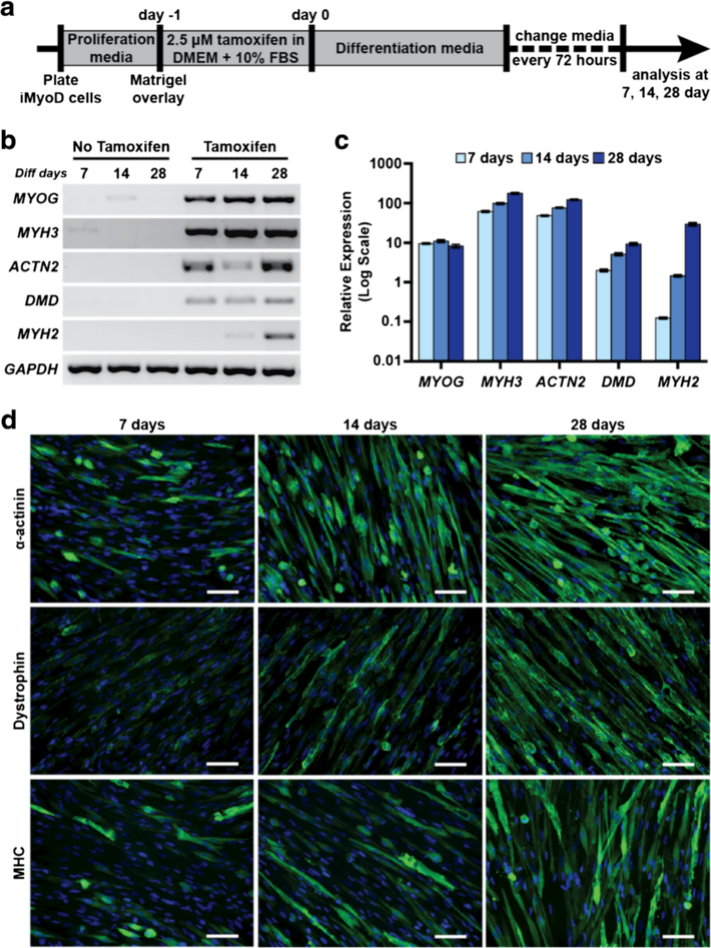
Myogenic differentiation and maturation of induced iMyoD UDCs. **a** Timeline for differentiation of iMyoD-transduced cells. **b** RT-PCR for myogenic maturation markers in iMyoD-transduced line 2, with or without tamoxifen (2.5 μM, 24 h), and culture in differentiation media as indicated (7, 14, and 28 days). Myogenin (*MYOG*) and embryonic myosin heavy chain (*MYH3*) were the earliest markers detected. **c** Real-time semi-quantitative PCR results demonstrated that dystrophin (*DMD*) and adult myosin heavy chain (*MYH2*) were detected with further differentiation. **d** Images of myogenic markers in iMyoD-transduced line 2. Increased muscle marker expression over time was accompanied by myotube elongation. Nuclei (*blue*); *Scale bar* = 100 μm

We assessed myotube differentiation on alternative coated surfaces including collagen, laminin, fibronectin, or poly-lysine. We also assessed the effectiveness of Matrigel overlay. We found that matrigel overlay of cells on collagen I-treated plates prior to tamoxifen induction offered the most robust differentiation into myotubes based on morphological appearance. Matrigel overlay was applied when cells reached confluence, and this was followed by tamoxifen induction in DMEM with 10 % FBS (2.5 μM, 24–48 h), and culture in HD media for the indicated time course. The outline for this optimized protocol is depicted in Fig. 3a.

MyoD controls temporal expression of early and late genes in muscle development through a feed-forward mechanism [33]. To characterize myogenic marker expression, we tested tamoxifen-treated iMyoD cells by RT-PCR. Robust and sustained expression of *MYOG* (myogenin) and *MYH3* (embryonic myosin) was observed (Fig. 3b). In addition, there was a temporal increase in expression of *ACTN2* (α-actinin) and *DMD* (dystrophin) (Fig. 3b). Expression of *MYH2*, the adult form of skeletal myosin, was only detected when differentiation was extended to the 28d time point (Fig. 3b). Similarly, real-time PCR detected most transcripts after 7 days of differentiation, with sustained or moderately increased expression over time (Fig. 3c). However, *DMD* and *MYH2* demonstrated greater temporal increases in transcript expression as myogenic maturation progressed (Fig. 3c). Evaluation of myotubes by immunofluorescence microscopy showed accumulation of α-actinin, dystrophin, and fast myosin heavy chain (MHC), concordant with the messenger RNA (mRNA) data. Expression of these muscle proteins was associated with increased myotube formation, organization, and elongation, indicative of myogenic differentiation and maturation over time (Fig. 3d).

**Fig. 4:**
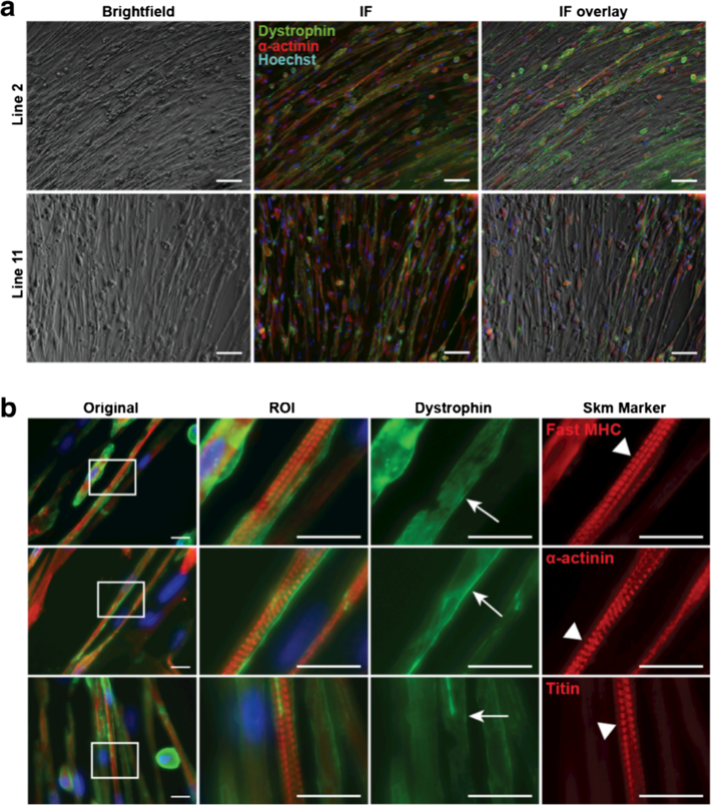
Maturation of myotubes at 28-day and 35-day time points. **a** Bright-field images of myotube formation at the 28-day time point in iMyoD lines 2 and 11 after induction and differentiation (*left panels*). *Middle panels* show images of dystrophin (*green*) and α-actinin (*red*) protein expression, taken in the same field as the bright-field panels. The *right panels* show the merged images revealing dystrophin and α-actinin expressions throughout the lengths of the myotubes. *Scale bar* = 100 μm. **b** Images of iMyoD-induced UDCs after 35 days of differentiation showing expression of fast MHC, α-actinin, and titin. The magnified region of interest (ROI) is outlined in the first panels (*white rectangles*). *White arrows* highlight the membrane localization of dystrophin (*green*). *White arrowheads* highlight the formation of sarcomeres (*red*). *Scale bar* = 25 μm

To further characterize iMyoD-directed myogenic maturation of urine cells, we analyzed reprogrammed cultures differentiated for 28 and 35 days. Low-magnification bright-field images of tamoxifen-treated iMyoD lines 2 and 11 demonstrated elongated myotube formation after 28 days in HD differentiation media (Fig. 4a) in which expression of α-actinin and dystrophin was observed throughout each fiber (Fig. 4a). Spontaneously twitching myotubes were observed in a subset of fields at both 28 and 35 days of culture, indicating sarcomere formation and function (Additional files 1 and 2: Movies S1 and S2). Sarcomere formation was confirmed with expression of fast myosin, α-actinin, and titin, each localized internally to sarcolemmal dystrophin expression and in the expected pattern (Fig. 4b). These results demonstrated efficient myogenic reprogramming, differentiation, and maturation of iMyoD-transduced urine cells.

Additional file 1:
**Movie 1.** Video showing spontaneous myotube twitching. (MOV 1366 kb)

Additional file 2:
**Movie 2.** Video showing spontaneous myotube twitching. (MOV 1869 kb)

### Direct reprogramming of urine cells to model muscular dystrophy

**Fig. 5:**
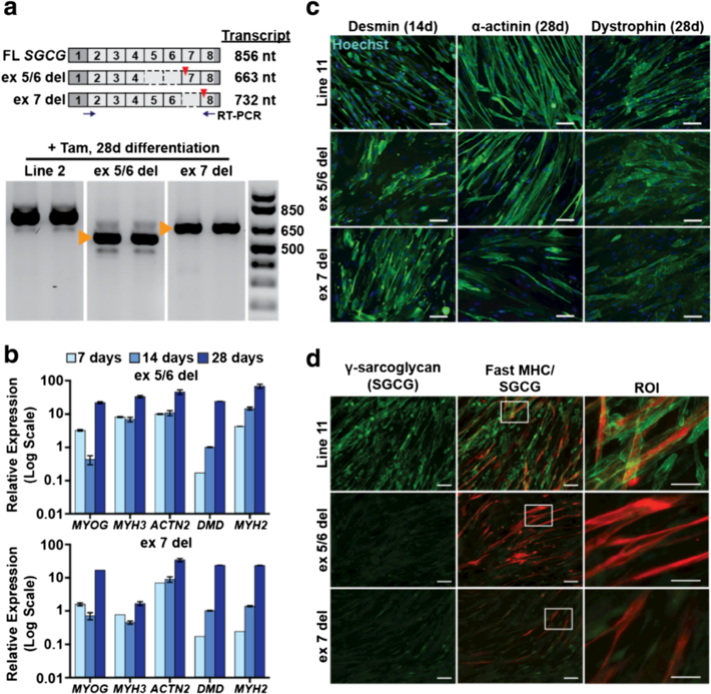
Myogenic reprogramming of LGMD2C UDCs. **a** Schematic showing exon organization of the γ-sarcoglycan (*SGCG*) mRNA transcript and two LGMD2C patients with frameshifting deletions affecting the *SGCG* locus that lead to premature stop codons (*red triangles*). One has a deletion of exons 5 and 6 (ex5/6del), and the other has a deletion of exon 7 (ex7del). RT-PCR demonstrated *SGCG* transcripts from UDCs after treatment with tamoxifen (2.5 μM, 24 h) and culture in differentiation media for 28d. *Orange arrowheads* indicate the mutant transcripts lacking the expected exons. **b** Real-time semi-quantitative PCR results demonstrate myogenic reprogramming of ex5/6del (*top*) and ex7del (*bottom*) after treatment with tamoxifen (2.5 μM, 24 h) and culture in differentiation media as indicated (7, 14, and 28 days). **c** Images of myogenic markers in iMyoD control and LGMD2C ex5/6del and ex7del patient-derived lines. LGMD2C patient-derived cells formed myotubes comparable to those of control cells. Nuclei (*blue*); *Scale bar* = 50 μm. **d** Desmin, α-actinin, dystrophin, and fast MHC were expressed in LGMD2C cells. γ-sarcoglycan (*green*) was not detected. *White boxes* outline double-labeled regions of interest (ROI) enlarged in *far right panels*. Images show γ-sarcoglycan expression and localization to the membrane in line 11, as opposed to no detectable expression of γ-sarcoglycan in LGMD2C patient myotubes. *Scale bar* = 100 μm in *left* and *middle panels*; 50 μm in *right panels*

The iMyoD reprogramming strategy was applied to urine cells derived from two patients with Limb Girdle Muscular Dystrophy type 2C (LGMD2C), which results from loss of function mutations in the gene encoding γ-sarcoglycan (SGCG) (Fig. 5a). Urine-derived cells from each patient were successfully transduced with the iMyoD lentivirus. Real-time PCR analysis of tamoxifen-treated iMyoD LGMD2C cells demonstrated successful reprogramming into the myogenic lineage (Fig. 5b) associated with morphological myotube formation (Fig. 5c). RT-PCR analysis demonstrated full-length *SGCG* transcript expression in reprogrammed control iMyoD cells and truncated mutant *SGCG* transcript expression in the reprogrammed iMyoD patient cells (Fig. 5a). Sarcolemmal associated γ-sarcoglycan protein was observed in control but not in LGMD2C cells, consistent with their mutation status (Fig. 5d).

**Fig. 6:**
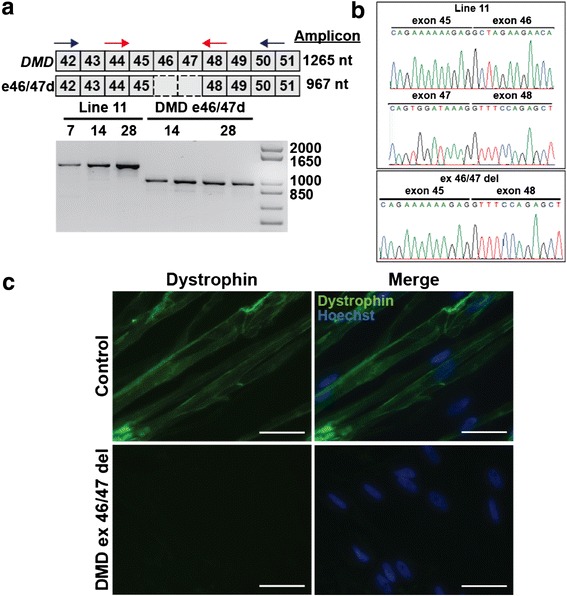
Myogenic reprogramming of DMD patient-derived urine cells. **a** Schematic showing organization of dystrophin exons 42 to 51 (*top*) and DMD patient with a frameshift deletion of exons 46 and 47 (*bottom*). RT-PCR demonstrated DMD transcript expression from iMyoD-transduced line 11 control and DMD ex46/47del mutant cell lines, after tamoxifen induction (2.5 μM, 24 h) and differentiation as indicated. *Blue arrows* indicate the location of the outer primers that produce the PCR products in the gel below. *Red arrows* indicate nested primers used to generate transcripts used for sequencing. **b** Sequencing results from RT-PCR products from iMyoD-reprogrammed urine-derived cells. The sequenced transcripts were generated from a set of nested primers (*red*) shown in **a**. Line 11 showed the expected exon45-exon46 and exon47-exon48 junctions. The ex46/47del DMD individual shows the out-of-frame mutant exon45-exon48 junction. **c** Immunofluorescence images of iMyoD line 17 and DMD ex46/47del cells labeled with dystrophin using the NCL-DYSB antibody to dystrophin. The antibody to dystrophin showed membrane localization of dystrophin in control cells (*top*). No dystrophin was detected in DMD patient-derived myotubes. *Scale bar* = 50 μm

We applied the same iMyoD strategy to DMD urine cells. The DMD patient harbored a frameshift deletion of exons 46 and 47 (ex46/47del) (Fig. 6a). RT-PCR analysis demonstrated full-length *DMD* transcript expression in reprogrammed control iMyoD cells and truncated *DMD* mutant transcript expression in the reprogrammed iMyoD patient cells (Fig. 6a). Sequence analysis of the PCR-amplified transcripts confirmed the deletion of exons 46 and 47 in the reprogrammed patient-derived cells (Fig. 6b). Sarcolemma-associated dystrophin protein was observed in control cells (Fig. 6c). In contrast, dystrophin protein was not detected in reprogrammed DMD patient cells after 28d differentiation (Fig. 6c). Thus, the iMyoD strategy in urine cells replicated the primary LGMD and DMD phenotypes in vitro.

### CRISPR/Cas9 mediated genome editing in single urine cell clones

**Fig. 7:**
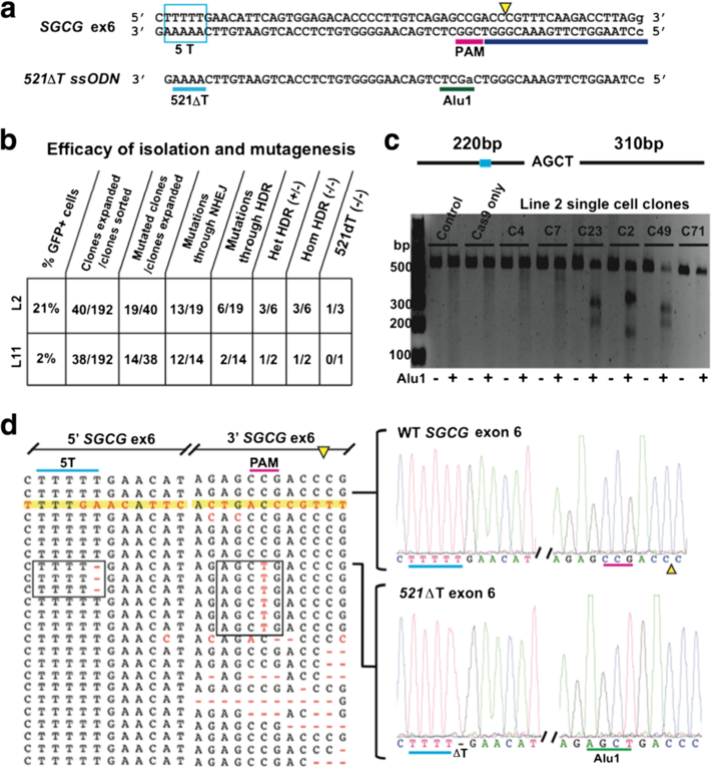
CRISPR/Cas9-mediated gene editing in UDCs. **a** Targeting strategy to introduce the 521ΔT point mutation into control *SGCG* exon 6 through gRNA guided, Cas9 nuclease generated double strand DNA break (DSB) with ssODN-mediated HDR repair. A portion of the *SGCG* exon 6 coding region is shown; a single T was removed from the 5T sequence region (*cyan box*). The downstream gRNA target sequence is highlighted in *blue*, the PAM sequence in *pink*, and the Cas9 DSB indicated by a *yellow triangle*. Below it is a portion of the 142 bp ssODN HDR repair template encoding the single T deletion along with a downstream G > A synonymous mutation (*green*) that destroys the PAM sequence and introduces an Alu1 restriction site. **b** Summary of efficiency of CRISPR/Cas9 protocol. **c** Alu1 restriction digest of a PCR-amplified genomic region including *SGCG* exon 6 and the flanking intronic sequences. **d** *Left*, partial summary of the Sanger sequencing results from individual line 2 edited clones. *Right*, the DNA trace from an unedited control and a clone with a homozygous deletion of a single T from the 5T of *SGCG* exon 6, recreating the most common LGMD2C mutation

Finally, we evaluated CRISPR (clustered regularly interspaced short palindromic repeats)/Cas9 (CRISPR-associated protein 9) mediated genome editing in urine cells. For these studies, the goal was to introduce the most common LGMD2C disease-causing mutation into control cell lines by deleting one thymine from *SGCG* exon 6 to disrupt the reading frame [26]. To generate the 521ΔT mutation, we used a gRNA targeting downstream of five sequential thymine residues and a single-stranded oligonucleotide (ssODN) to mediate homology-directed repair (HDR). The ssODN targeting the minus strand contained a synonymous G > A transition to disrupt the protospacer adjacent motif (PAM) and introduce a novel Alu1 restriction site (Fig. 7a). CRISPR components were electroporated into control UDC lines 2 and 11, followed by FACS single cell sorting for GFP into 96-well plates. Approximately 20 % of the clones derived from single sorted cells grew sufficiently so that they could be evaluated for CRISPR-mediated gene editing (Fig. 7b). The proliferation capacity of FACS-sorted clones was similar in untreated cells and cells electroporated with the Cas9 GFP plasmid alone.

The genomic region including *SGCG* exon 6 was PCR amplified to analyze the results of gene editing. Restriction digest of the PCR products revealed the presence of an Alu1 site in a subset of clones, indicative of HDR repair (Fig. 7c). Sequence analysis demonstrated efficient genome editing in UDCs with the expected variety of both heterozygous and homozygous mutations, including insertions and deletions from nonhomologous end joining (NHEJ) repair and specific point mutations directed by HDR (Fig. 7c, d). Generation of clones with both heterozygous and homozygous 521ΔT mutations was evident in line 2 (Fig. 7c, d). Interestingly, a subset of HDR-edited clones contained a point mutation in the PAM region without the upstream thymine deletion, suggesting that this region is refractory to editing and/or required a gRNA target closer to the 5T region. A summary of the gene editing results is shown in Fig. 7b, Additional file 3: Table S1 and S2, and Figure S1 and S2. Collectively, these data demonstrated the ability to edit the genome of urine-derived cells with CRISPR/Cas9, generating single cell clones with a specific point mutation.

## Discussion

The limited availability of human skeletal muscle tissue encumbers the study of muscle development and the pathology of muscle disease. Many novel strategies have been taken to overcome this limitation, relying on genetic engineering with direct and indirect reprogramming of cells. Regardless of strategy, a key component to success is the initial cell source, specifically the ease of acquisition and ability to reprogram. Urine is an underutilized, noninvasive, and cost effective source of primary human cells that has served as a platform for urothelial-based culture and iPSC reprogramming [19–21]. Recent advances in urine cell isolation have generated clonal populations of autologous human cells with a high proliferative capacity, evident telomerase activity, and plasticity for reprogramming into multiple lineages [20, 21]. These characteristics make urine-derived cells an attractive source of primary human cells for in vitro models. We now extended these findings showing that inducible expression of MyoD efficiently reprogrammed UDCs into the myogenic lineage, providing a simple strategy to generate an in vitro model of skeletal muscle from virtually any human subject.

Urine cells demonstrate an inclination towards the mesodermal lineage. A subpopulation of cells, classified as urine stem cells, express pericyte and mesenchymal stem cell markers and were able to differentiate into podocytes, smooth muscle, and urothelial cells [20, 21, 32]. The ability of UDCs to differentiate into skeletal muscle is less defined. Studies showed growth on specific biomaterials, presence of specific growth factors, or defined media modifications promoted expression of myogenic markers such as MyoD, desmin, and myosin [23, 34]. However, these cells were not observed to form striations nor to display contractile properties. Given the known expression of these markers, it is possible that without sufficient MyoD, UDCs differentiate more towards smooth rather than striated muscle lineages [21]. The strategy with an induced MyoD pulse combined with media manipulation drove urine cells through the initial stages of myogenic development into a more mature phenotype, complete with expression of dystrophin, adult myosin, titin, and γ–sarcoglycan, along with the presence of functional sarcomeres. Despite these advancements, this cellular model still lacks major components of mature muscle such as transverse tubules and resident stem cell niches.

An alternative strategy to direct reprogramming with MyoD would be to first reprogram UDCs into iPSCs and then use the iPSC lines for myogenic differentiation. This approach has the advantage of iPSC self-renewal, although immortalization of UDCs directly could obviate this need in vitro. DMD UDCs were previously successfully reprogrammed into iPSC and differentiated into cardiomyocytes, recapitulating cardiac phenotypes in vitro [4].

**Fig. 8:**
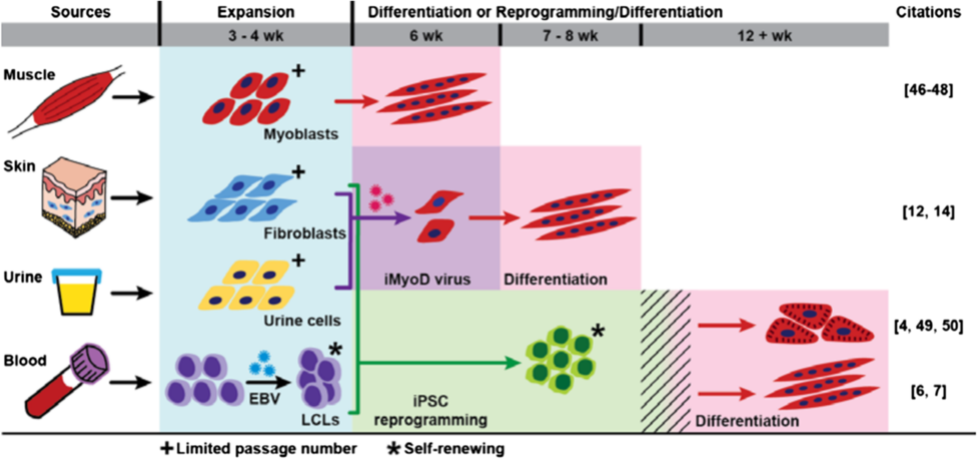
Overview of options for modeling skeletal muscle disease in cell culture. Primary cell sources for establishing cell cultures include the muscle, skin, urine, and blood. Myoblasts cultured from muscle biopsies require approximately 3 to 4 weeks for expansion and an additional 2 weeks of culture for myotube differentiation [46–48]. Primary dermal fibroblasts isolated from skin biopsies require 3 to 4 weeks for expansion. Fibroblasts can then be transduced with inducible MyoD (iMyoD) lentivirus, expanded, and differentiated to yield myotubes by approximately 8 weeks [12, 14]. Urine cells require 3 to 4 weeks for expansion and a similar time frame for iMyoD-induced myotube formation as dermal fibroblasts. Lymphocytes isolated from blood are typically transformed using Epstein-Barr virus (EBV) to generate lymphoblastoid cell lines (LCLs), which requires 3 to 4 weeks. Fibroblasts, urine-derived cells, and LCLs can all be reprogrammed to induced pluripotent stem cells (iPSCs). Once generated and expanded, iPSCs will be subjected to quality control measures such as immunofluorescence for pluripotency markers, karyotyping, and embryoid body formation or teratoma formation for three germ layers. As each laboratory has its own quality control measures, this process will add variable amount of time for iPSC culture (indicated by *hatch marks*). iPSCs can be readily differentiated into cardiomyocytes [4, 49, 50]. Improved efficiency for myotube differentiation from iPSCs has been reported, but additional advances are expected to enhance the efficiency of this process [6, 7]

Skeletal muscle differentiation of iPSCs has proven more challenging, although more efficient skeletal muscle differentiation from iPSC using small molecules has recently been reported [7]. Interestingly, the ease of urine cell isolation, transduction with iMyoD virus, and reprogramming into iPSC suggest an ability to employ multiple parallel models for the study of muscle disease. A summary of cell types for modeling muscle disease and the timeline for generating these models in culture is shown in Fig. 8.

We also showed that UDCs can be directly gene edited using CRISPR/Cas9 technology. The use of gene editing for muscle disease has focused intently on genetic correction strategies with significant success on the study of muscular dystrophy, as recent studies showed the ability to correct DMD disease-causing mutations in animal models and human cells [35–43]. However, the ability to insert mutations of interest is also useful since it allows the study of disease models without the need to access patients directly. Nevertheless, gene editing in human iPSCs is challenged by the availability of patient cell lines, isolation of pure clonal populations, and low editing efficiency, especially when targeting precise mutations with homology-dependent repair [29, 44, 45]. While some technical aspects of the CRISPR/Cas9 system are still undergoing improvement, the relative ease of urine cell isolation, coupled with the ability to generate single cell clones, makes this an attractive primary cell platform for CRISPR/Cas9 gene editing.

## Conclusions

Here, we showed that direct reprogramming of urine cells using MyoD is feasible from patients with muscle disease and can recapitulate the basic disease phenotype. Because urine cells are readily available and collected noninvasively, this approach provides a highly useful model of human muscle disease.

## Additional files

Additional file 3: Table S1. Sequencing results for line 2 clones. **Table S2.** Sequencing results for line 11 clones. **Figure S1.** Example of sequencing results from CRISPR/Cas9 edited urine line 2. **Figure S2.** Example of sequencing results from CRISPR/Cas9 edited urine line 11. (PDF 1694 kb)

## Abbreviations

UDCs: Urine-derived cells
iMyoD: Inducible MyoD
FBS: Fetal bovine serum
DMEM: Dulbecco’s Modified Eagle Medium
PBS: Phosphate-buffered saline
FGF: Fibroblast growth factor
PDGF: Platelet-derived growth factor
EGF: Epidermal growth factor
RT-PCR: Reverse transcription polymerase chain reaction
CRISPR: Clustered regularly interspaced short palindromic repeats
Cas9: CRISPR-associated protein 9
ssODN: Single-stranded oligodeoxynucleotide
PAM: Protospacer adjacent motif
gRNA: Guide RNA
HDR: Homology-dependent repair
NHEJ: Nonhomologous end joining
DSB: Double strand DNA break
FACS: Fluorescence-activated cells sorting
RFLP: Restriction fragment length polymorphism
LGMD: Limb girdle muscular dystrophy
DMD: Duchenne muscular dystrophy
SGCG: γ-Sarcoglycan
MHC: Myosin heavy chain
iPSCs: Induced pluripotent stem cells
SEM: Standard error of the mean
HD: Hydrocortisone dexamethasone
