# Supplementary material for: Direct reprogramming of urine-derived cells with inducible MyoD for modeling human muscle disease
Source: Skelet Muscle. 2016 Sep 15;6:32. doi: 10.1186/s13395-016-0103-9 (PMC5025576; doi:10.1186/s13395-016-0103-9)
Supplement: Additional file 3: Table S1. — Sequencing results for line 2 clones. Table S2. Sequencing results for line 11 clones. Figure S1. Example of sequencing results from CRISPR/Cas9 edited urine line 2. Figure S2. Example of sequencing results from CRISPR/Cas9 edited urine line 11. (PDF 1694 kb) [file 13395_2016_103_MOESM3_ESM.pdf]

Supplemental table 1. Sequencing results for line 2 clones

clone	*SGCG* exon 6 sequence	genotype
C4	GGCCTGAAGGGGCTCTTTTTGAACATTCAGTGGAGACACCCCTTGTCAGAGCCGACCCGT-TTCAAGACCTTAG	WT
C7	GGCCTGAAGGGGCTCTTTTTGAACATTCAGTGGAGACACCCCTTGTCAGAGCCGACCCGT-TTCAAGACCTTAG	WT
C11	GGCCTGAAGGGGCTCTTTTTGAACATTCAGTGGAGACACCCCTTGTCAGAGCCGACCCGT-TTCAAGACCTTAG	WT
C13	GGCCTGAAGGGGCTCTTTTTGAACATTCAGTGGAGACACCCCTTGTCAGAGCCGACCCGT-TTCAAGACCTTAG	WT
C14	GGCCTGAAGGGGCTCTTTTTGAACATTCAGTGGAGACACCCCTTGTCAGAGCCGACCCGT-TTCAAGACCTTAG	WT
C15	GGCCTGAAGGGGCTCTTTTTGAACATTCAGTGGAGACACCCCTTGTCAGAGCCGACCCGT-TTCAAGACCTTAG	WT
C17	GGCCTGAAGGGGCTCTTTTTGAACATTCAGTGGAGACACCCCTTGTCAGAGCCGACCCGT-TTCAAGACCTTAG	WT
C20	GGCCTGAAGGGGCTCTTTTTGAACATTCAGTGGAGACACCCCTTGTCAGAGCCGACCCGT-TTCAAGACCTTAG	WT
C26	GGCCTGAAGGGGCTCTTTTTGAACATTCAGTGGAGACACCCCTTGTCAGAGCCGACCCGT-TTCAAGACCTTAG	WT
C28	GGCCTGAAGGGGCTCTTTTTGAACATTCAGTGGAGACACCCCTTGTCAGAGCCGACCCGT-TTCAAGACCTTAG	WT
C30	GGCCTGAAGGGGCTCTTTTTGAACATTCAGTGGAGACACCCCTTGTCAGAGCCGACCCGT-TTCAAGACCTTAG	WT
C31	GGCCTGAAGGGGCTCTTTTTGAACATTCAGTGGAGACACCCCTTGTCAGAGCCGACCCGT-TTCAAGACCTTAG	WT
C42	GGCCTGAAGGGGCTCTTTTTGAACATTCAGTGGAGACACCCCTTGTCAGAGCCGACCCGT-TTCAAGACCTTAG	WT
C51	GGCCTGAAGGGGCTCTTTTTGAACATTCAGTGGAGACACCCCTTGTCAGAGCCGACCCGT-TTCAAGACCTTAG	WT
C57	GGCCTGAAGGGGCTCTTTTTGAACATTCAGTGGAGACACCCCTTGTCAGAGCCGACCCGT-TTCAAGACCTTAG	WT
C59	GGCCTGAAGGGGCTCTTTTTGAACATTCAGTGGAGACACCCCTTGTCAGAGCCGACCCGT-TTCAAGACCTTAG	WT
C61	GGCCTGAAGGGGCTCTTTTTGAACATTCAGTGGAGACACCCCTTGTCAGAGCCGACCCGT-TTCAAGACCTTAG	WT
C62	GGCCTGAAGGGGCTCTTTTTGAACATTCAGTGGAGACACCCCTTGTCAGAGCCGACCCGT-TTCAAGACCTTAG	WT
C65	GGCCTGAAGGGGCTCTTTTTGAACATTCAGTGGAGACACCCCTTGTCAGAGCCGACCCGT-TTCAAGACCTTAG	WT
C66	GGCCTGAAGGGGCTCTTTTTGAACATTCAGTGGAGACACCCCTTGTCAGAGCCGACCCGT-TTCAAGACCTTAG	WT
C71	GGCCTGAAGGGGCTCTTTTTGAACATTCAGTGGAGACACCCCTTGTCAGAGCCGACCCGT-TTCAAGACCTTAG	WT
C23	GGCCTGAAGGGGCTCTTTT-GAACATTCAGTGGAGACACCCCTTGTCAGAGCTGACCCGT-TTCAAGACCTTAG	HDR,521dT -/-
C41	GGCCTGAAGGGGCTCTTTT-GAACATTCAGTGGAGACACCCCTTGTCAGAGCTGACCCGT-TTCAAGACCTTAG	HDR,521dT +/-
C48	GGCCTGAAGGGGCTCTTTT-GAACATTCAGTGGAGACACCCCTTGTCAGAGCTGACCCGT-TTCAAGACCTTAC	HDR,521dT +/-
C26	GGCCTGAAGGGGCTCTTTTTGAACATTCAGTGGAGACACCCCTTGTCAGAGCTGACCCGT-TTCAAGACCTTAG	HDR, no 521dT* +/-
C47	GGCCTGAAGGGGCTCTTTTTGAACATTCAGTGGAGACACCCCTTGTCAGAGCTGACCCGT-TTCAAGACCTTAG	HDR, no 521dT* -/-
C49	GGCCTGAAGGGGCTCTTTTTGAACATTCAGTGGAGACACCCCTTGTCAGAGCTGACCCGT-TTCAAGACCTTAG	HDR, no 521dT* -/-
C2	GGCCGATGGGGTCTTTTTGAACATTCTCGGAAACACCCCTTGTCAGAACTGACCCGTTTCAAGAACTTAGGTAA	insert, missense
C40	GACCGGAATGGGCTCTTTTTGAACATTCAGAGGACACACCCCTTGTCACACCCGACCCGCATTTTAGGAAAAAT	insert, missense
C21	GGCCTGAAGGGGCTCTTTTTGAACATTCAGTGGAGACACCCCTTGTCAGAGCCGACC--T-TTCAAGACCTTAG	2bp deletion, +/-
C22	GGCCTGAAGGGGCTCTTTTTGAACATTCAGTGGAGACACCCCTTGTCAGAGCCGA-CCGT-TTCAAGACCTTAG	1bp deletion, -/-
C29	GGCCTGAAGGGGCTCTTTTTGAACATTCAGTGGAGACACCCCTTGTCAGAGCCGAC------------------	16bp deletion, -/-
C33	GGCCTGAAGGGGCTCTTTTTGAACATTCAGTGGAGACACCCCTTGTCAGAGCCG-----------AGACCTTAG	10bp deletion, -/-
C36	GGCCTGAAGGGGCTCTTTTTGAACATTCAGTGGAGACACCCCTTGTCAGAGCCGACC----TTCAAGACCTTAG	3bp deletion, -/-
C44	GGCCTGAAGGGGCTCTTTTTGAACATTCAGTGGAGACACCCCTTGTCAGAGCCGACCCG------AGACCTTAG	5bp deletion, -/-
C63	GGCCTGAAGGGGCTCTTTTTGAACATTCAGTGGAGACACCCCTTGTCAGAGCCGACCC---------------G	14bp deletion, -/-
C69	GGCCTGAAGGGGCTCTTTTTGAACATTCAGTGGAGACACCCC-----A-AG---ACC--T-T--A---C----G	deletion, +/-
C1	GGCCTGAAGGGCTTCTTTTTGAACCTTCAGTGGAC-CCCCTCTTGTCACAGAC--CCCCT-TTG-AGACCTTAG	deletion, missense
C32	GGCCTGAAAGGGCTCTTTTTGAACATTCATTGGAAACACCCCTT--------------GT-TTCAAGACCTTAC	deletion, missense
C37	GGCCTGGGGGGGCTCTTTTTGAACATTCGGTGGAGACACCCCTTGTCAGAG---AC--G-----A---CC--A-	deletion, missense

* C→T PAM mutation indicates HDR, but cannot rule out synonymous NHEJ mutation due to lack of 521dT

Supplemental table 2. Sequencing results for line 11 clones

clone	*SGCG* exon 6 sequence*	genotype
**C3**	GGCCTGAAGGGGCTCTTTTTGAACATTCAGTGGAGACACCCCTTGTCAGAGCCGACC--C-GTTTCAAGACCTTAG	**WT**
**C10**	GGCCTGAAGGGGCTCTTTTTGAACATTCAGTGGAGACACCCCTTGTCAGAGCCGACC--C-GTTTCAAGACCTTAG	**WT**
**C13**	GGCCTGAAGGGGCTCTTTTTGAACATTCAGTGGAGACACCCCTTGTCAGAGCCGACC--C-GTTTCAAGACCTTAG	**WT**
**C15**	GGCCTGAAGGGGCTCTTTTTGAACATTCAGTGGAGACACCCCTTGTCAGAGCCGACC--C-GTTTCAAGACCTTAG	**WT**
**C18**	GGCCTGAAGGGGCTCTTTTTGAACATTCAGTGGAGACACCCCTTGTCAGAGCCGACC--C-GTTTCAAGACCTTAG	**WT**
**C27**	GGCCTGAAGGGGCTCTTTTTGAACATTCAGTGGAGACACCCCTTGTCAGAGCCGACC--C-GTTTCAAGACCTTAG	**WT**
**C28**	GGCCTGAAGGGGCTCTTTTTGAACATTCAGTGGAGACACCCCTTGTCAGAGCCGACC--C-GTTTCAAGACCTTAG	**WT**
**C30**	GGCCTGAAGGGGCTCTTTTTGAACATTCAGTGGAGACACCCCTTGTCAGAGCCGACC--C-GTTTCAAGACCTTAG	**WT**
**C31**	GGCCTGAAGGGGCTCTTTTTGAACATTCAGTGGAGACACCCCTTGTCAGAGCCGACC--C-GTTTCAAGACCTTAG	**WT**
**C33**	GGCCTGAAGGGGCTCTTTTTGAACATTCAGTGGAGACACCCCTTGTCAGAGCCGACC--C-GTTTCAAGACCTTAG	**WT**
**C37**	GGCCTGAAGGGGCTCTTTTTGAACATTCAGTGGAGACACCCCTTGTCAGAGCCGACC--C-GTTTCAAGACCTTAG	**WT**
**C39**	GGCCTGAAGGGGCTCTTTTTGAACATTCAGTGGAGACACCCCTTGTCAGAGCCGACC--C-GTTTCAAGACCTTAG	**WT**
**C41**	GGCCTGAAGGGGCTCTTTTTGAACATTCAGTGGAGACACCCCTTGTCAGAGCCGACC--C-GTTTCAAGACCTTAG	**WT**
**C46**	GGCCTGAAGGGGCTCTTTTTGAACATTCAGTGGAGACACCCCTTGTCAGAGCCGACC--C-GTTTCAAGACCTTAG	**WT**
**C50**	GGCCTGAAGGGGCTCTTTTTGAACATTCAGTGGAGACACCCCTTGTCAGAGCCGACC--C-GTTTCAAGACCTTAG	**WT**
**C55**	GGCCTGAAGGGGCTCTTTTTGAACATTCAGTGGAGACACCCCTTGTCAGAGCCGACC--C-GTTTCAAGACCTTAG	**WT**
**C56**	GGCCTGAAGGGGCTCTTTTTGAACATTCAGTGGAGACACCCCTTGTCAGAGCCGACC--C-GTTTCAAGACCTTAG	**WT**
**C57**	GGCCTGAAGGGGCTCTTTTTGAACATTCAGTGGAGACACCCCTTGTCAGAGCCGACC--C-GTTTCAAGACCTTAG	**WT**
**C59**	GGCCTGAAGGGGCTCTTTTTGAACATTCAGTGGAGACACCCCTTGTCAGAGCCGACC--C-GTTTCAAGACCTTAG	**WT**
**C61**	GGCCTGAAGGGGCTCTTTTTGAACATTCAGTGGAGACACCCCTTGTCAGAGCCGACC--C-GTTTCAAGACCTTAG	**WT**
**C62**	GGCCTGAAGGGGCTCTTTTTGAACATTCAGTGGAGACACCCCTTGTCAGAGCCGACC--C-GTTTCAAGACCTTAG	**WT**
**C63**	GGCCTGAAGGGGCTCTTTTTGAACATTCAGTGGAGACACCCCTTGTCAGAGCCGACC--C-GTTTCAAGACCTTAG	**WT**
**C65**	GGCCTGAAGGGGCTCTTTTTGAACATTCAGTGGAGACACCCCTTGTCAGAGCCGACC--C-GTTTCAAGACCTTAG	**WT**
**C66**	GGCCTGAAGGGGCTCTTTTTGAACATTCAGTGGAGACACCCCTTGTCAGAGCCGACC--C-GTTTCAAGACCTTAG	**WT**
**C6**	GGCCTGAAGGGGCTCTTTTTGAACATTCAGTGGAGACACCCCTTGTCAGAGCTGACC--C-GTTTCAAGACCTTAG	**HDR +/-,no 521dT****
**C47**	GGCCTGAAGGGGCTCTTTTTGAACATTCAGTGGAGACACCCCTTGTCAGAGCTGACC--C-GTTTCAAGACCTTAG	**HDR +/-,no 521dT****
**C32**	GGCCTGAAGGGGCTCTTTTTGAACATTCAGTGGAGACACCCCTTGTCAAAGCCGACCCGC-GTTTCAAGACCTTAG	**2bp insertion, +/-**
**C42**	GGCCTGAAGGGGCTCTTTTTGAACATTCAGTGGAGACACCCCTTGTCAAAGCCGACCCGC-GTTTCAAGACCTTAG	**2bp insertion, +/-**
**C45**	GGCCTGAAGGGGCTCTTTTTGAACATTCAGTGGAGACACCCCTTGTCAGAGCCGACCTTAGGTTTCAAGACCTTAG	**4bp insertion, -/-**
**C17**	GGCCTGAAGGGGCTCTTTTTGAACATTCAGTGGAGACACCCCTTGTCAGAGCCGA-C--C-GTTTCAAGACCTTAG	**1bp deletion, -/-**
**C26**	GGCCTGAAGGGGCTCTTTTTGAACATTCAGTGGAGACACCCCTTGTCAGAGCCGA-C--C-GTTTCAAGACCTTAG	**1bp deletion, -/-**
**C35**	GGCCTGAAGGGGCTCTTTTTGAACATTCAGTGGAGACACCCCTTGTCAGAGCCGA-C--C-GTTTCAAGACCTTAG	**1bp deletion, -/-**
**C38**	GGCCTGAAGGGGCTCTTTTTGAACATTCAGTGGAGACACCCCTTGTCAGAGCCGA-C--C-GTTTCAAGACCTTAG	**1bp deletion, -/-**
**C36**	GGCCTGAAGGGGCTCTTTTTGAACATTCAGTGGAGACACCCCTTGTCAGAG-------------------CCTTAG	**16bp deletion, -/-#**
**C64**	GGCCTGAAGGGGCTCTTTTTGAACATTCAGTGGAGACACCCCTTGTCAGAGCCGACCTT---TCTCAACACTTTGT	**indel/missense, +/-**

* 35/38 contiguous sequences aligned, 3 did not align

** C→T PAM mutation indicates HDR, but cannot rule out synonymous NHEJ mutation due to lack of 521dT

noisy trace requires sub-cloning for homozygous mutation confirmation

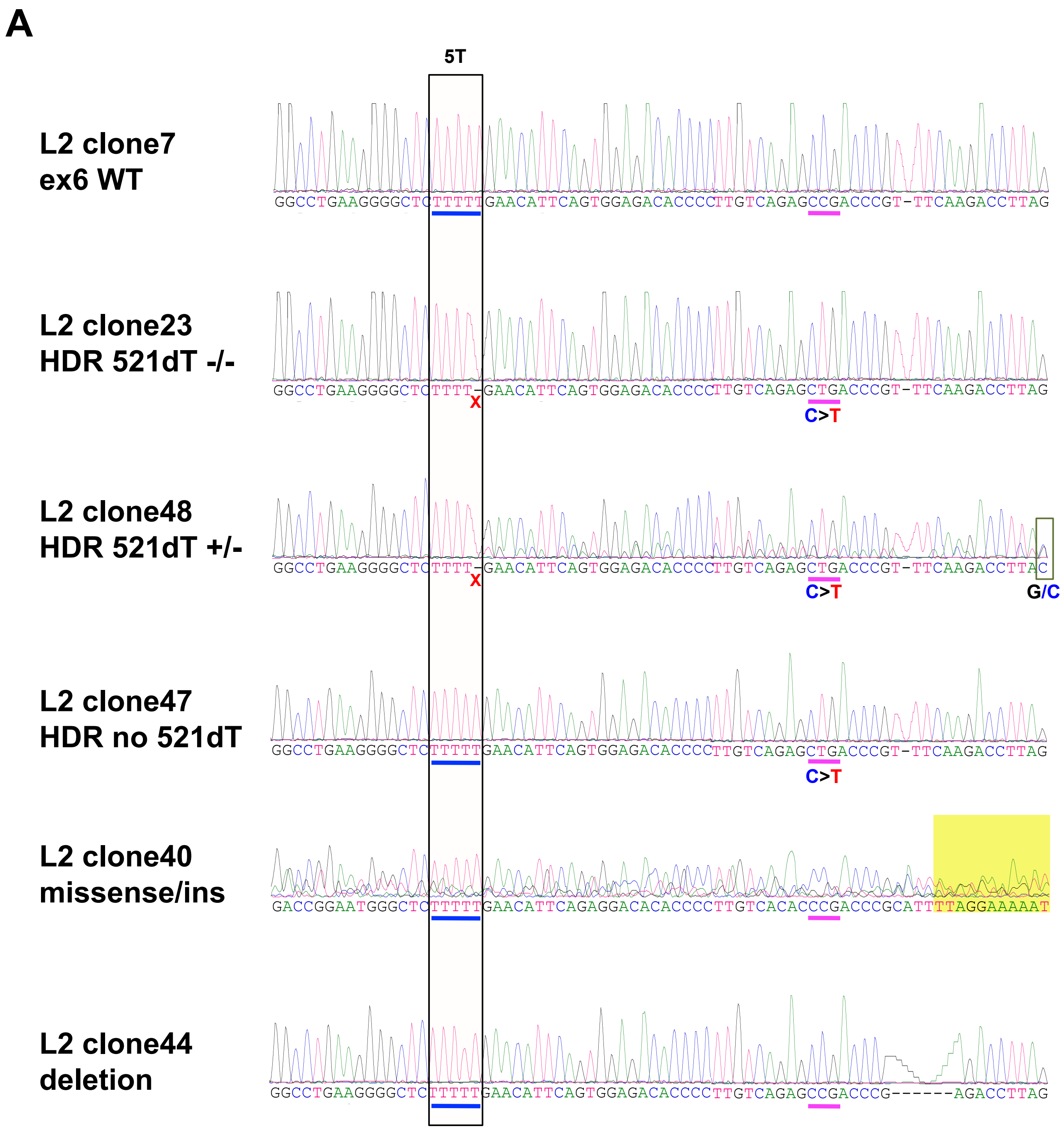

Supplementary Figure 1. Example of sequencing results from CRISPR/Cas9 edited urine line 2. Cells were electroporated with the Cas9 GFP plasmid, the PCR amplified U6 gRNA construct and the 521ΔT ssODN, then single cell FACS sorted for GFP expression. **A)** Representative traces demonstrating the different genotypes observed including WT, heterozygous and homozygous deletions of 521T, HDR repair without the 521ΔT mutation, and NHEJ generated indels.

A

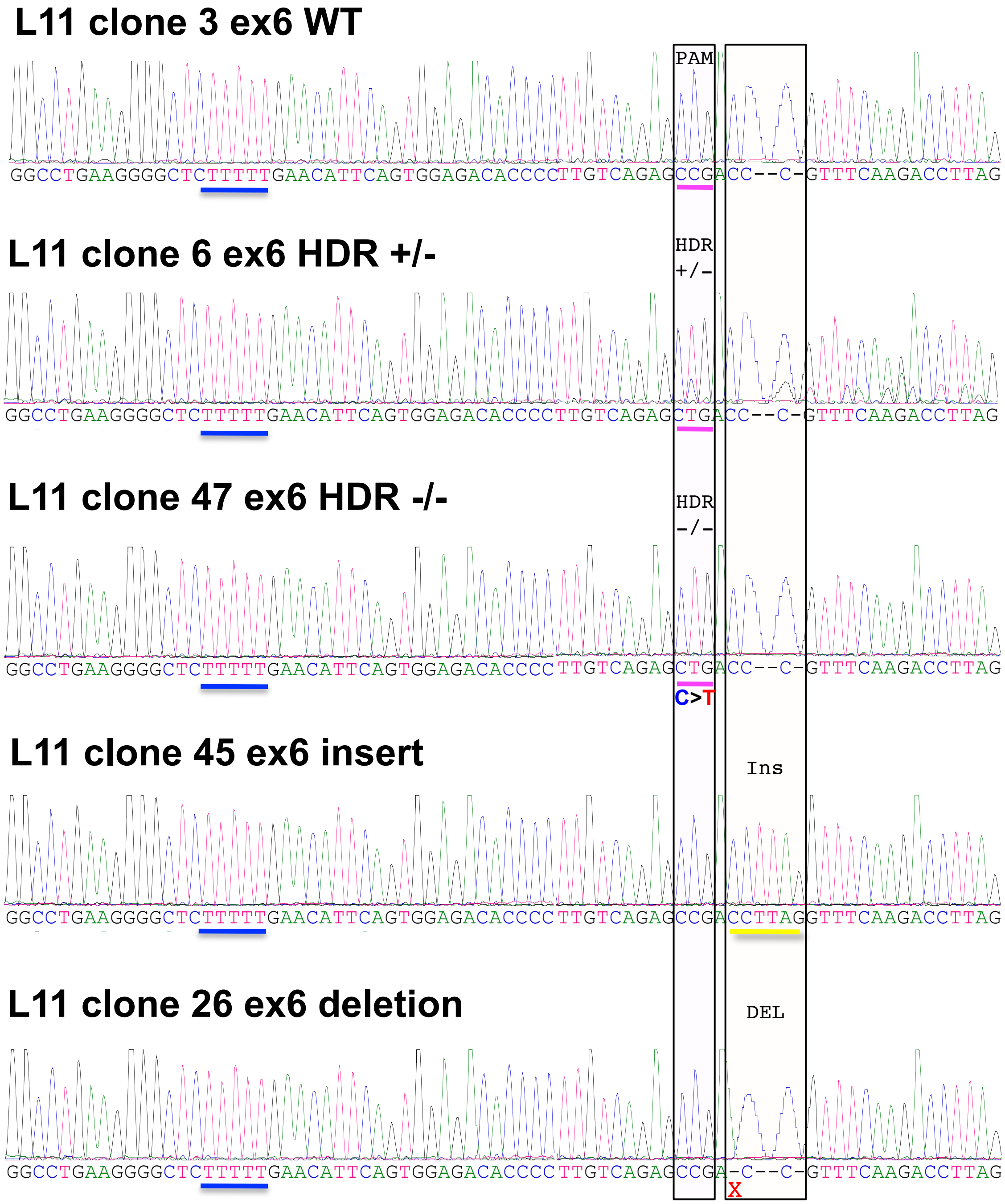

Supplementary Figure 2. Example of sequencing results from CRISPR/Cas9 edited urine line 11. Cells were electroporated with the Cas9 GFP plasmid, the PCR amplified U6 gRNA construct and the 521ΔT ssODN, then single cell FACS sorted for GFP expression. **A)** Representative traces demonstrating the different genotypes observed including WT, heterozygous and homozygous deletions of 521T, HDR repair without the 521ΔT mutation, and NHEJ generated indels.
